# Comparative Operational Performance of Baited Logs, Lure Traps, and Flight-Intercept Traps in a Province-Scale Surveillance Program for Pine Wood-Boring Beetles in Yunnan, Southwestern China

**DOI:** 10.3390/insects17050526

**Published:** 2026-05-20

**Authors:** Jidong Liu, Qi Jiang, Shaoshun He, Zhengqing Wu, Jianrong Wu, Taoyou Ping, Yujie Liu

**Affiliations:** 1 Pest Research Center, Yinglin Branch, Yunnan Institute of Forest Inventory and Planning, Kunming 650032, China; 13708400927@163.com (J.L.);; 2 Linxiang District Forestry and Grassland Pest Control and Quarantine Station, Lincang 677000, China

**Keywords:** pine wood-boring beetles, broad-spectrum surveillance, lure traps, baited logs, flight-intercept traps

## Abstract

National and provincial surveillance of pine wood-boring pests in China is designed to detect damaging taxa, map occurrence, assess risk and loss, and support early warning, zoning and control decisions. Province-scale comparisons of alternative monitoring devices remain rare, especially under the operational conditions required by such programs in climatically and topographically heterogeneous forests. Using data from the 2025–2026 systematic survey of pine wood-boring pests in Yunnan Province, China, we integrated several monitoring datasets to compare baited logs, lure traps, and flight-intercept traps. The harmonized database comprised 2603 standard monitoring subcompartments and 3519 installed sites, including 4080 baited-log piles, 4807 lure-trap units, and 373 flight-intercept traps. Main performance analyses focused on active sites with at least one collection event (570 baited-log sites, 63 flight-intercept sites, and 496 lure-trap sites), whereas installed site summaries were retained to characterize operational coverage. Because the study was observational and the three devices have different sampling mechanisms, we interpreted detection probability as the primary early warning metric, and catch, operational taxon richness, standardized yield, and cost metrics as supporting indicators of diagnostic and operational return. Site-level comparisons were complemented with paired analyses of 21 co-located subcompartments, a more comparable subset defined within county × elevation band × host group strata represented by all three methods, county-clustered regression, and a taxonomic-resolution sensitivity analysis. Lure traps consistently had the highest detection probability (0.73), the greatest cumulative catch (8617 individuals), and the broadest operational taxonomic coverage (45 operational taxa). In county-clustered models, lure traps had higher odds of detection (odds ratio = 11.25, 95% CI: 5.64–22.43) and higher catch rates (incidence rate ratio = 5.97, 95% CI: 2.26–15.76) than baited logs after adjustment for elevation band, host group, and collection effort. The same ranking persisted in the more comparable subset and after exclusion of unresolved family-, subfamily-, genus-, and unknown-level records. Standardized yield peaked at 1500–2200 m. Scenario-based costing showed that lure traps had the lowest cost per captured and resolved captured individual, whereas detection cost estimates were interpreted together with absolute detections and operational taxonomic output. Overall, the results support a tiered surveillance architecture in which lure traps serve as the primary routine early warning tool, baited logs provide targeted complementary information, and flight-intercept traps are reserved mainly for exploratory or faunistic surveys.

## Simple Summary

Province-wide surveillance for pine wood-boring beetles supports forest resource security, biosecurity, early warning, and precision pest management rather than faunistic inventory alone. Such programs need surveillance tools that can detect pine wood-boring beetles early, work across heterogeneous mountain landscapes, and remain practical for routine field deployment. Using data from the 2025–2026 systematic survey of pine wood-boring pests in Yunnan Province, China, we compared baited logs, lure traps, and flight-intercept traps under real operational conditions. We then examined whether the ranking among methods held in paired subcompartments, in a more comparable county–elevation–host subset, and after excluding coarsely identified records. Across all checks, lure traps remained the strongest primary early warning option; they detected beetles at more sites, captured more individuals, and recorded more operational taxa than the other two methods. Baited logs remained useful when host use needed to be confirmed or immature material was required, but they were markedly less efficient for routine surveillance. Flight-intercept traps contributed least to early warning within this monitoring framework and are better suited to specialized biodiversity or faunistic surveys. Monitoring returns were generally highest at mid-elevations. Taken together, the results support a tiered strategy for mountainous pine forests: use lure traps as the main routine biosecurity and precision pest-management tool, retain baited logs for targeted diagnostic follow-up, and reserve flight-intercept traps for specialized exploratory surveys.

## 1. Introduction

Pine wood-boring beetles are among the most consequential disturbance agents in conifer forests because they weaken tree vigor, accelerate mortality, alter stand structure, and often interact with fungi, nematodes, or other associated organisms that amplify damage across landscapes [1,2,3,4,5,6,7]. In pine systems, the surveillance problem is therefore broader than tracking a single named pest. Effective early warning is a forest biosecurity and pest management function: it must detect both known damaging taxa and a wider assemblage of accompanying xylophagous beetles that may signal declining stand resistance, host stress, or incipient outbreaks before visible crown decline becomes widespread. From a management perspective, surveillance also provides the baseline evidence needed to estimate damage, judge risk, prioritize regions, and select proportionate prevention and control measures.

Trap-based surveillance has therefore become a core component of forest insect biosecurity because it can reveal beetle occurrence earlier and more consistently than symptom-based inspection alone [1,2,8]. The published literature has established that trap performance is not a fixed property of a device, but a context-dependent outcome shaped by trap architecture, panel area, color, surface treatment, cup design, lure composition and release rate, trap height, and microhabitat placement [1,2,9,10,11,12,13,14,15,16,17,18,19,20,21]. For applied surveillance, these variables matter because agencies need methods that convert limited field visits into reliable detections and interpretable taxonomic evidence.

Recent studies on generic surveillance have expanded the field beyond single-species optimization. Multi-component lures and landscape-aware sampling designs can detect diverse native and alien bark- and wood-boring beetles at ports of entry, in forests, and across heterogeneous landscapes [8,22,23,24,25,26]. These studies provide a strong foundation for broad-spectrum monitoring, but they also highlight that performance depends on target guild, landscape setting, attractant blend, trap design, and the management endpoint being optimized [1,2,24,25,26]. A device that performs well in a controlled trial or biodiversity survey may not necessarily provide the best early warning return in a routine provincial monitoring program.

In China, this question is especially important because pine wood-boring beetles are embedded in the broader biosecurity context of pine wilt disease and other decline syndromes. The biology and seasonal activity of *Monochamus alternatus* and *M. saltuarius*, together with the documented carrier status of multiple beetle species for *Bursaphelenchus xylophilus*, increase the management value of surveillance tools that are sensitive, standardized, scalable, and usable under rugged terrain, uneven staffing, and non-uniform revisit intervals [21,25,27,28].

On the basis of this literature, the knowledge gap addressed by the present study is operational rather than purely methodological. First, most published evaluations examine one target species, one trap modification, or a limited set of sites, so it remains unclear whether the advantages of semiochemical-baited traps persist across a province-scale mountain surveillance network [1,2,7,15,16,17,19,24,26]. Second, few studies compare semiochemical lure traps, baited host material, and passive flight-intercept traps within the same administrative program, although these are the alternatives that management agencies must choose among when balancing detection probability, taxonomic breadth, labor demand, repeatability, and cost. Third, published comparisons often emphasize capture efficiency or faunistic yield, whereas routine early warning requires decision-oriented endpoints such as realized field effort, site-level detection probability, operational taxonomic output, robustness under common support strata, and cost per usable unit of information. These gaps are important because operational surveillance programs must select methods that remain effective not only in experimental trials but also under heterogeneous terrain, uneven revisit frequency, variable host composition, and real administrative constraints.

Yunnan is a particularly informative region in which to close this gap. Its pine forests span strong gradients in elevation, thermal regime, moisture seasonality, land use, and accessibility, and they contain extensive *Pinus yunnanensis* Franch. forests together with other pine hosts. *P. yunnanensis* is emphasized here because it is the dominant and most operationally important pine host in much of Yunnan, provides the main matrix for provincial pine wood-borer surveillance, and overlaps with important shoot- and wood-boring beetles such as *Tomicus yunnanensis* Kirkendall & Faccoli [29,30,31]. A monitoring method that retains its advantage across this heterogeneous setting is more informative for mountainous pine regions than a method that performs only under narrow local conditions.

China’s 2025–2026 systematic survey of pine wood-boring pests created a rare opportunity to evaluate this question under real operational conditions. The program was launched as a damage-oriented survey to clarify pest composition, distribution, occurrence severity, natural enemy resources, loss, risk, zoning, and regional control priorities [29]. Within this framework, the technical scheme recognized lure traps, baited logs, and flight-intercept traps as alternative or complementary broad-spectrum monitoring devices in healthy standard subcompartments [29]. The resulting Yunnan dataset combines large spatial coverage with programmatic heterogeneity in deployment intensity, revisit frequency, host context, and terrain, making it suitable for comparative evaluation of surveillance performance rather than only trap mechanics.

The novelty of this study therefore lies in translating published trap surveillance principles into a province-scale, decision-oriented comparison of three device types under real implementation conditions. Rather than testing a single new trap design, we integrate deployment, collection event, and taxonomic records from 2603 standard monitoring subcompartments; distinguish installed coverage from active sampling efforts; use site-level detection probability as the primary early warning endpoint; and test whether device rankings persist in paired subcompartments, common support strata, county-clustered models, and taxonomic resolution sensitivity analyses. We further interpret performance jointly with elevation, host context, deployment density, and scenario-based cost, thereby linking surveillance output to the practical design of monitoring architectures.

Accordingly, our objectives were to: (i) quantify monitoring coverage and realized field effort for baited logs, lure traps, and flight-intercept traps; (ii) compare detection probability, cumulative catch, operational taxon richness, and effort-standardized yield among methods; (iii) examine context dependence with respect to elevation, host group, and deployment density; and (iv) assess relative cost-effectiveness under routine provincial surveillance. Because the database was observational rather than experimentally balanced, we interpret the contrasts as operational differences in realized surveillance performance, not as fully causal estimates of intrinsic trap efficiency. We expected lure traps to provide the strongest routine early warning performance because semiochemical attraction increases the probability of intercepting dispersing adults, whereas baited logs and flight-intercept traps were expected to provide narrower but complementary diagnostic or exploratory information.

## 2. Materials and Methods

### 2.1. Study Framework and Data Sources

This study drew on the Yunnan component of the 2025–2026 systematic survey dataset for pine wood-boring pests and the associated technical documentation [29]. The national technical scheme was explicitly damage-oriented and combined ground inspection, trap monitoring, remote sensing checks, and laboratory detection or identification through a monitoring platform and mobile application [29]. Within this broader pest survey framework, we analyzed the device-based records relevant to pine wood-boring beetle surveillance. The standard subcompartment dataset included 2603 broad-spectrum monitoring subcompartments and provided the administrative and stand attributes used for linkage, including prefecture, county, township, village, land-use category, stand condition, area, elevation, and dominant host information. Three additional method-specific datasets contained records for lure traps, baited logs, and flight-intercept traps, including deployment information, collection event records, and taxonomic identification records.

**Figure 1:**
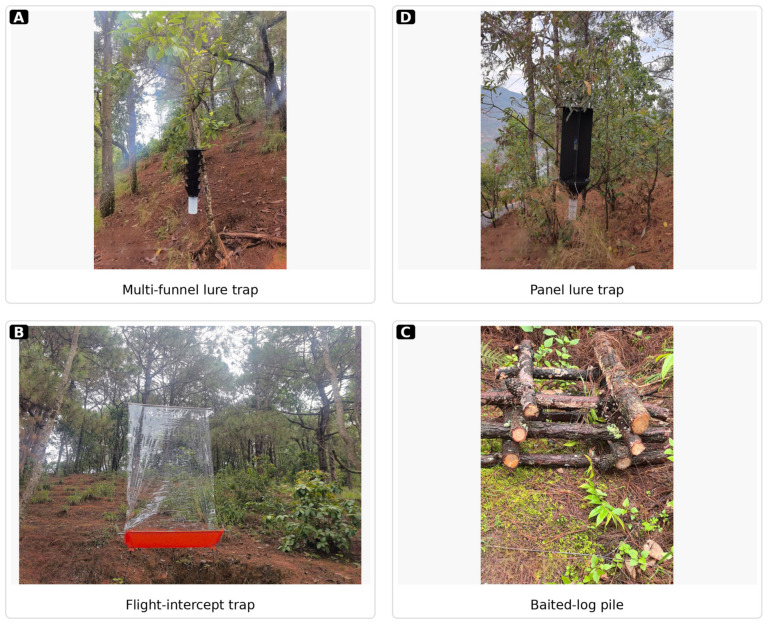
Representative field photographs of the monitoring devices used in the Yunnan monitoring program: (**A**) multi-funnel lure trap; (**B**) flight-intercept trap; (**C**) baited-log pile; and (**D**) panel lure trap.

Consistent with the national scheme, the three devices represented different surveillance logics and were therefore interpreted as complementary operational tools rather than mechanically equivalent traps [29]. Lure traps relied on semiochemical attraction and were intended primarily to intercept dispersing adults. Baited logs used freshly prepared host material to simulate attractive breeding substrate and therefore reflected a host-mediated attraction and colonization process. Flight-intercept traps acted passively by intercepting insects in flight without an active odor plume. This functional contrast was central to the interpretation of the study: detection probability was considered the primary early warning endpoint, whereas abundance, operational taxon richness, standardized yield, and cost metrics were treated as secondary indicators of diagnostic return and operational efficiency. Because the monitoring network was implemented under a single province-wide program, the database allowed comparison under realistic operational heterogeneity rather than tightly controlled small-plot experiments. Representative field photographs of the monitoring devices used in the field program are shown in Figure 1.

**Figure 2:**
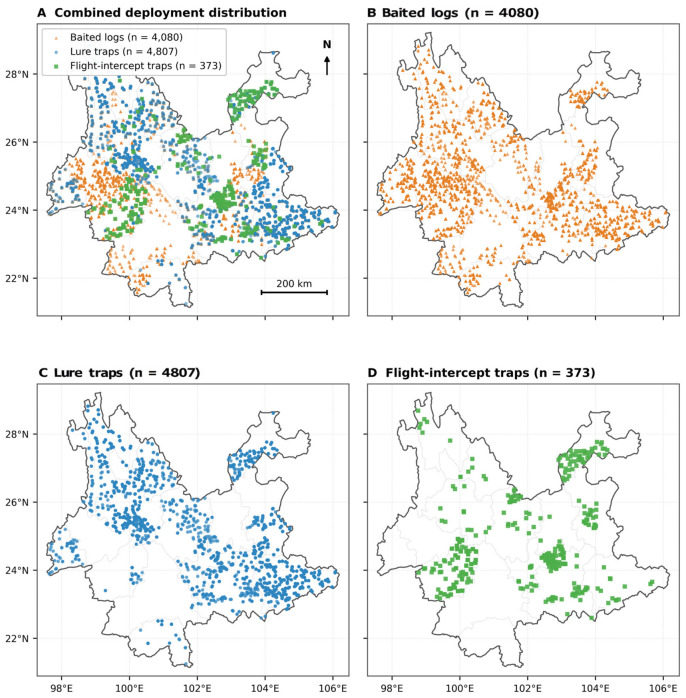
Province-scale distribution of baited logs, lure traps, and flight-intercept traps across Yunnan Province, Southwestern China. Points represent unique deployment units extracted from the deployment records of the three device-specific datasets. County boundaries are shown in light gray and the provincial outline in dark gray.

To summarize the province-scale spatial allocation of surveillance effort, we prepared a descriptive distribution map of monitoring devices across Yunnan Province (Figure 2). Geographic coordinates were extracted only from the deployment records of the three device-specific datasets because the standard subcompartment dataset did not contain longitude or latitude fields. The mapped points therefore represent unique deployment units of baited logs, lure traps, and flight-intercept traps rather than all standard monitoring subcompartments. For cartographic display, the provincial outline and county boundaries of Yunnan were drawn from a public GeoJSON boundary dataset and overlaid with the extracted device coordinates.

### 2.2. Device Specifications, Specimen Recovery, and Identification

Lure traps consisted of multi-funnel or panel traps fitted with replaceable commercial lure cores for pine wood-boring beetles. The provincial operational records identified the lure products as broad-spectrum pine wood-borer lures rather than as a single experimental formulation. Where product labels or technical sheets were available to field teams, the active attractant components included pine host volatiles such as α-pinene, β-pinene, ethanol, and 3-carene, together with the longhorn beetle aggregation pheromone 2-undecyloxy-1-ethanol for *Monochamus*-oriented deployments. Manufacturer-specific carriers, exact release rates, and lure age at each service visit were not consistently retained in the administrative database; consequently, lure chemistry was described at the active component level and was not modeled as an independently randomized treatment factor. Because both multi-funnel and panel traps were used operationally, “lure trap” was analyzed as the program’s semiochemical-baited surveillance category rather than as a single standardized trap architecture; trap architecture was therefore not modeled separately.

Baited-log monitoring followed the national technical scheme for the systematic survey of pine wood-boring pests [29], and the present analysis retained the beetle records generated by this device. In selected healthy pine subcompartments, three piles were generally established from local pine hosts, mainly *Pinus yunnanensis*, with other local *Pinus* species used where dominant. Each pile consisted of approximately ten freshly cut 1.0–1.5 m bolts from healthy or weakened trees without visible borer attack; bark was retained, excess branches were removed, and bolts were placed in sunny microsites, usually in crisscross piles. Deployment was conducted mainly from April to October. Logs were inspected on day 3 and then in approximately 7-day intervals; one to two bolts per pile were dissected at each visit, and remaining material was dissected or reared when adult emergence was needed for identification. Specimens were preserved in absolute ethanol and labelled with pile ID, location, and date. Each pile was treated as one installed unit, whereas bolt diameter, exact felling-to-deployment interval, and realized exposure duration were not recorded consistently and are acknowledged as reproducibility limitations.

Specimens from lure traps and flight-intercept traps were removed from collecting containers at each service visit, transferred to labelled collection vials or containers, and linked to the corresponding subcompartment, device, and collection event record. Baited-log specimens were collected during scheduled inspections by opening or disassembling piles; examining bark surfaces, cut ends, and galleries; and removing adults or immature stages directly from the material. When immature specimens could not be identified reliably, specimens or infested material were retained for further examination or field cage/net rearing to adult emergence when feasible; however, the administrative database did not contain a consistent field that separated direct extraction from rearing-through records. Identifications were made primarily from adult morphology by trained county, township, and provincial taxonomic personnel using the national technical scheme and regional diagnostic references, with difficult records checked at higher technical levels before harmonization. All records were then assigned to the finest reliable taxonomic category supported by the evidence.

### 2.3. Data Harmonization and Analytical Unit

All method-specific records were harmonized by a unique subcompartment code (i.e., 53090201301100415). Deployment records were used to quantify site coverage, numbers of deployed units, coordinates, elevation, and local host descriptors. Collection event records were used to measure revisit effort, whereas taxonomic identification records were aggregated to obtain abundance and operational taxon richness. We distinguished between installed sites and active sites. Installed sites were defined as subcompartments with at least one deployment record. Active sites were defined as deployed subcompartments with at least one collection event, regardless of whether any target beetles were detected.

For coverage analyses, all installed sites were retained. For performance analyses, inference was restricted to active sites so that zero catches represented realized sampling rather than missing field effort. The harmonized database contained 1745 baited-log sites, 1413 lure-trap sites, and 361 flight-intercept sites. The active site subset contained 570 baited-log sites, 496 lure-trap sites, and 63 flight-intercept sites. Total installed units were 4080 baited-log piles, 4807 lure-trap units, and 373 flight-intercept traps, with 3012, 6011, and 164 collection events, respectively. Where a subcompartment contained multiple units of the same method, captures were summed to the subcompartment-by-method level, while the number of deployed units and revisit counts was retained for effort standardization. The installed-to-active transition was also treated as an informative operational descriptor because it reflected whether deployed devices subsequently generated realized sampling effort.

**Figure 3:**
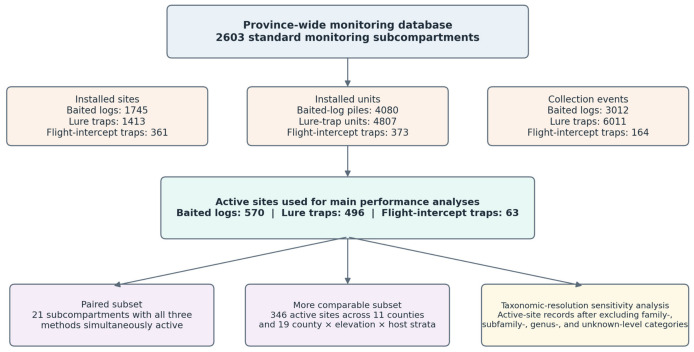
Construction of the main analytical subsets used in the comparative evaluation. The schematic distinguishes the province-wide database, installed sites and effort summaries, active sites used for the main performance analyses, the 21 paired subcompartments, the more comparable subset of 346 active sites, and the taxonomic resolution sensitivity analysis subset.

Figure 3 summarizes how the main analytical subsets were derived. From the province-wide database, we distinguished standard monitoring subcompartments, installed sites, active sites, the 21 paired subcompartments in which all three methods were simultaneously active, and the more comparable subset of 346 active sites distributed across 11 counties and 19 county × elevation band × host group strata. This schematic was included to show clearly how the operational coverage data related to the inferential subsets used in the comparative analyses.

### 2.4. Response Metrics

Five operational response variables were calculated and interpreted according to surveillance objective. First, detection probability was defined as the proportion of active sites with at least one recorded pine wood-boring beetle taxon and was treated as the primary early warning metric. Second, cumulative catch per site represented the total number of individuals recorded across all collection visits within a site. Third, operational taxon richness per site represented the number of distinct operational taxa recorded for that site. These abundance- and richness-based metrics were interpreted as indicators of diagnostic information and follow-up value rather than as direct estimates of beetle density. Fourth, captures per collection event were used as a routine efficiency indicator that reflected field return per service visit. Fifth, standardized capture yield was calculated as the number of individuals/(deployed units × collection events), thereby allowing comparison among methods even when the number of devices or revisit frequencies differed.

An operational taxon was defined as the finest reliable category recorded in the official dataset after field or laboratory verification. This category could be a species-level record, such as *M. alternatus* Hope or *Pissodes yunnanensis* Langor & Zhang; a genus-level record, such as *Pissodes* spp.; a higher rank record, such as *Cerambycidae* spp., *Scolytinae* spp., or *Curculionidae* spp.; or an unresolved operational category when no finer diagnosis could be justified from the evidence. Richness in the main analysis was therefore interpreted as operational taxon richness rather than standardized species-level richness. This choice was appropriate for comparing surveillance performance under real monitoring conditions, but it could overstate or understate species-level diversity if taxonomic resolution differed among methods. To test whether mixed taxonomic resolution altered the main ranking, we repeated selected comparisons after excluding records identified only as unknown, family-, subfamily-, or genus-level categories.

### 2.5. Contextual Variables

Yunnan spans steep climatic gradients over short distances. Because the operational datasets did not provide measured site-level temperature or precipitation, elevation was used as the primary climatic proxy. Active sites were grouped into three elevation bands: low (<1500 m), mid (1500–2200 m), and high (>2200 m). Habitat context was represented by land-use type from the standard subcompartment dataset, and host context was represented by dominant host descriptors from the standard and method-specific datasets. For multivariate modeling, host categories were simplified into *P. yunnanensis* Franch., *P. armandii* Franch., *P. kesiya* var. *langbianensis* (A.Chev.) Gaussen (Simao pine), *P. densata* Mast. (high-mountain pine), and other or mixed hosts.

### 2.6. Robustness, Common Support, and Interpretation Framework

The national technical scheme emphasized data traceability, standardized reporting, and integration of device records with subcompartment-level stand information [29]. In the present analysis, the subcompartment was retained as the core operational unit. This prevented sites with several lure traps or baited-log piles from being treated as several independent ecological replicates.

Because the program was not experimentally balanced, we used two additional checks to improve comparability. First, we defined a common support subset by retaining only strata in which all three methods were represented within the same county × elevation band × host group combination. This yielded 346 active sites distributed across 11 counties and 19 strata. Second, we fitted county-clustered regression models to reduce the influence of within-county dependence and unequal implementation intensity. These steps improve comparability but do not recreate a randomized design; residual confounding associated with local maintenance quality, lure age, accessibility, or background beetle pressure may still remain.

The interpretation framework was deliberately operational and objective-specific. We did not attempt to estimate absolute beetle density, emergence rate, breeding success, or intrinsic trap efficiency. Instead, we asked how much actionable surveillance information each method produced under the conditions in which provincial monitoring actually occurs. Because lure traps, baited logs, and passive intercept traps differ fundamentally in their sampling mechanisms, method comparisons were interpreted as comparisons of surveillance roles within one administrative architecture, not as proof that one physical trap design is universally superior. Accordingly, county-level deployment density was treated as a management proxy for crowding or concentration rather than as a mechanistic estimate of local trap interference.

### 2.7. Statistical Analysis

Differences in detection probability among methods were tested using a chi-square contingency analysis. Differences in cumulative catch, taxon richness, and captures per collection event were tested using Kruskal–Wallis tests followed by pairwise Mann–Whitney comparisons with Holm correction. A paired analysis was conducted for the subset of 21 subcompartments in which all three monitoring methods were simultaneously active; these contrasts were evaluated using Friedman tests.

To estimate contextual effects while controlling for effort, we fitted generalized estimating equation (GEE) logistic models for detection probability and negative-binomial generalized linear models for cumulative abundance with log (collection events) as an offset. County was treated as the clustering unit in the GEE models, an exchangeable working correlation structure was specified, and robust sandwich standard errors were reported to obtain conservative inference under uneven county-level implementation. The negative-binomial specification was used as a variance-robust model for strongly right-skewed, overdispersed surveillance counts. Monitoring method, elevation band, and host group were included as predictors. These models were interpreted as adjusted comparative effectiveness models rather than as causal treatment effect models because the database lacked direct weather measurements, stand density variables, independent indices of beetle pressure, and randomized deployment. For key model contrasts, odds ratios (ORs) or incidence rate ratios (IRRs) and 95% confidence intervals are reported. Analyses and figures were generated in Python using pandas, scipy, statsmodels, and matplotlib.

### 2.8. Scenario-Based Cost-Effectiveness Assessment

Cost-effectiveness was evaluated under three scenario bands representing low, base, and high operational expenditure per active site. The assumed costs were 200/280/360 CNY for baited-log monitoring, 100/140/180 CNY for flight-intercept monitoring, and 500/680/860 CNY for lure-trap monitoring. These estimates were used only as supporting sensitivity analyses of relative information cost, not as audited reconstructions of provincial expenditure. Because early warning surveillance prioritizes detection, we evaluated cost per detected active site as the scenario cost divided by detection probability. Cost per captured individual and cost per resolved captured individual were also calculated as secondary indicators of sampling return, but these metrics were not used alone to define operational superiority because they can favor methods that catch many individuals even if detection timing, diagnostic value, or target relevance differs.

## 3. Results

### 3.1. Monitoring Coverage and Realized Effort

**Figure 4:**
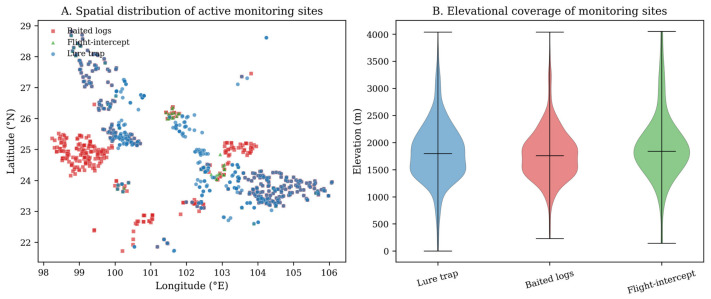
Spatial distribution and elevational coverage of active monitoring sites. Points represent active subcompartments by method, and the violin plot summarizes elevational coverage.

The Yunnan dataset showed a clear mismatch between nominal deployment and realized monitoring effort (Table 1; Figure 4). Baited logs were installed in the greatest number of subcompartments (1745), followed by lure traps (1413) and flight-intercept traps (361). Lure traps, however, accounted for the largest number of deployed units (4807) and the greatest servicing effort (6011 collection events). Baited logs comprised 4080 installed piles and 3012 collection events, whereas flight-intercept traps accounted for only 373 installed units and 164 collection events. Differences in the transition from installed to active status also suggest that operational feasibility varied among methods, rather than reflecting ecology alone.

When the analysis was restricted to active sites, the contrast among methods became more pronounced. We retained 570 active baited-log sites, 63 active flight-intercept sites, and 496 active lure-trap sites. Mean elevation was similar among methods (1796–1883 m), suggesting that all three were deployed across broadly comparable climatic space, even though county-level clustering differed. Most active sites were in arbor forest and were dominated by *P. yunnanensis*, indicating that the comparison primarily reflects operational surveillance in Yunnan pine landscapes rather than a balanced sample of all conifer systems in the province.

### 3.2. Overall Performance Comparison

**Figure 5:**
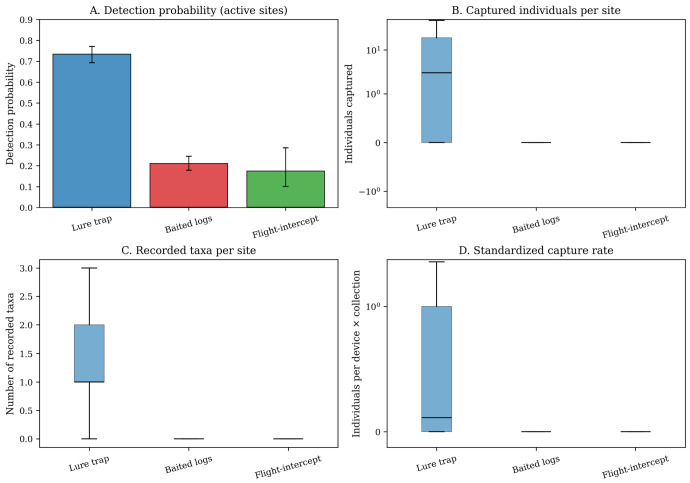
Performance metrics at active sites. (**A**) Detection probability; (**B**) cumulative catches per site; (**C**) operational taxon richness per site; and (**D**) standardized capture yield expressed as individuals per deployed unit × collection event. Panels (**B**,**D**) are shown on a pseudo-log scale with non-negative tick labels to accommodate zero-inflated and strongly right-skewed counts.

Performance differed sharply among methods (Table 2; Figure 5). Because detection probability was the primary early warning metric, the detection contrast provided the most direct evidence of routine surveillance performance. At active sites, detection probability was 0.73 for lure traps, compared with 0.21 for baited logs and 0.17 for flight-intercept traps (chi-square = 313.90, *p* < 0.001). Lure traps also produced far higher abundance-based returns, recording 8617 individuals versus 999 for baited logs and 44 for flight-intercept traps. Median cumulative catch per active site was three for lure traps but zero for the other two methods. Mean catch per collection event was 1.43 for lure traps, compared with 0.33 for baited logs and 0.27 for flight-intercept traps.

The same pattern was evident for operational taxonomic output. Lure traps recorded 45 operational taxa, whereas baited logs and flight-intercept traps recorded 18 and 6, respectively. Mean operational richness per active site was 1.45 for lure traps, compared with 0.26 for baited logs and 0.21 for flight-intercept traps. These values indicate broader diagnostic output in the operational records, but they should not be interpreted as unbiased estimates of community-level species richness because taxonomic resolution varied among records.

### 3.3. Paired and Robustness Analyses

**Figure 6:**
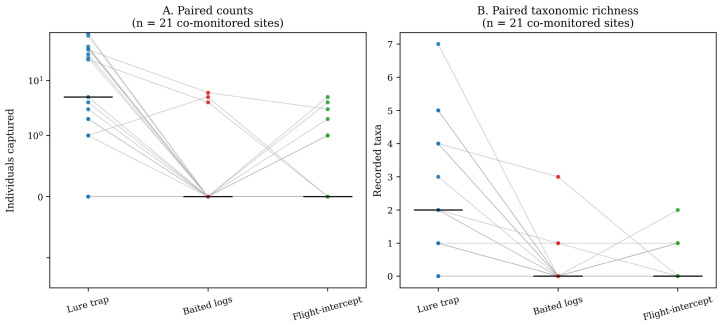
Paired comparison across the 21 subcompartments where all three methods were simultaneously active. Each line represents one subcompartment. Panel (**A**) is shown on a pseudo-log scale with non-negative tick labels to accommodate strongly right-skewed counts.

A paired comparison was possible for 21 subcompartments in which all three methods were simultaneously active (Figure 6). In this subset, mean cumulative catch was 20.29 individuals for lure traps, but only 0.71 for baited logs and 0.81 for flight-intercept traps. Mean taxon richness was 2.48, 0.24, and 0.38 taxa per site, respectively. Friedman tests confirmed significant among-method differences for both cumulative catch and richness, indicating that the advantage of lure traps was not simply a by-product of broad spatial placement.

To assess whether unequal representation among methods influenced the comparison, we repeated the analysis in the common support subset, which was restricted to 11 counties and 19 county × elevation × host strata represented by all three methods. The ranking did not change: detection probability was 0.784 for lure traps, 0.141 for baited logs, and 0.190 for flight-intercept traps, and total catches were 3340, 391, and 33 individuals, respectively. After adjustment for elevation band and host group, county-clustered GEE models showed that lure traps had 11.25-fold higher odds of detection than baited logs (95% CI: 5.64–22.43; *p* < 0.001), whereas flight-intercept traps did not differ significantly from baited logs. County-clustered negative-binomial models led to the same general conclusion for abundance, with lure traps producing 5.97-fold higher catch rates than baited logs (95% CI: 2.26–15.76; *p* < 0.001). These analyses strengthen the ranking observed in the main data, although some residual confounding may remain.

### 3.4. Taxonomic Composition, Complementarity, and Sensitivity to Taxonomic Resolution

**Figure 7:**
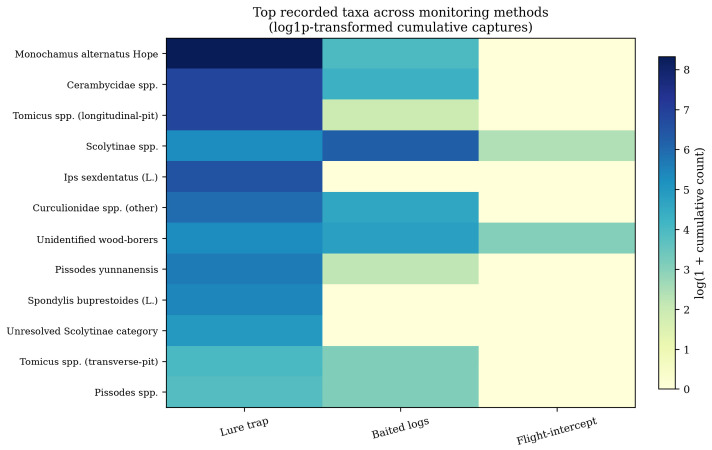
Heat map of dominant recorded taxa by monitoring method. Cell colors show log1p-transformed cumulative individual counts; underlying values are cumulative captures. Labels use scientific names where the operational record could be resolved, whereas higher rank or unresolved labels indicate categories retained because the administrative data did not support species-level assignment.

Catch composition further illustrated the functional differences among methods (Figure 7). Lure traps dominated captures of the major operational groups, including *M. alternatus* Hope, *Cerambycidae* spp., *Tomicus* spp. operational categories, *Ips sexdentatus* (L.), other *Curculionidae* spp., *Pissodes yunnanensis* Langor & Zhang, *Spondylis buprestoides* (L.), and *Pissodes* spp. In Figure 7, *Curculionidae* spp. denotes non-*Pissodes* weevil records that could not be resolved to species, whereas *Pissodes yunnanensis* and *Pissodes* spp. were retained as separate categories because they were recorded separately in the operational database. Baited logs yielded smaller catches overall, but they contained a relatively larger share of broadly identified Scolytinae and unknown wood-borer records, suggesting a useful supplementary role when host use confirmation or immature-stage association is needed. Flight-intercept traps produced the narrowest and sparsest taxonomic profile, with only low absolute abundance across a small number of categories.

The taxonomic resolution sensitivity analysis pointed in the same direction. After unresolved family-, subfamily-, genus-, and unknown-level records were excluded, lure traps still captured 6703 individuals from 26 resolved taxa, compared with 96 individuals from 5 resolved taxa for baited logs and 4 individuals from 1 resolved taxon for flight-intercept traps. The apparent dominance of lure traps was therefore not simply an artefact of broader coarse-level identification in the field records, although all richness-based results remain operational rather than complete community inventories.

### 3.5. Effects of Elevation, Host Context, and Deployment Density

**Figure 8:**
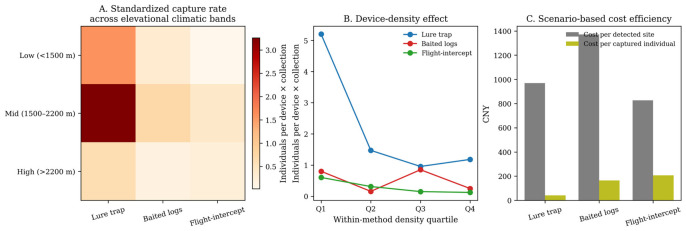
Context dependence and scenario-based cost efficiency. (**A**) Standardized yield across elevation bands; (**B**) within-method deployment density quartiles versus standardized yield; and (**C**) base scenario cost per detected active site and cost per captured individual.

Standardized capture yield showed a clear elevational pattern (Figure 8A). For all three methods, yields peaked in the 1500–2200 m band: 0.72 individuals per deployed unit × collection event for baited logs, 0.40 for flight-intercept traps, and 3.26 for lure traps. Yields were lower below 1500 m and dropped sharply above 2200 m, particularly for baited logs and lure traps. The county-clustered negative-binomial model indicated the same broad trend: after adjustment, lure traps had substantially higher catch rates than baited logs, whereas flight-intercept traps remained lower and statistically uncertain. Capture rate declined toward the high-elevation band, and sites dominated by *P. yunnanensis* tended to outperform the residual host category. County-level deployment density quartiles further suggested that lure-trap and baited-log yields were generally highest in the lowest-density quartile and became lower or more variable as clustering increased, consistent with diminishing returns under concentrated deployment.

### 3.6. Scenario-Based Cost-Effectiveness

Scenario-based costing supported the same operational interpretation but also showed why cost metrics should not be used alone (Figure 8C). Across the low, base, and high scenarios, the estimated cost per captured individual was 28.8, 39.1, and 49.5 CNY for lure traps, compared with 114.1, 159.8, and 205.4 CNY for baited logs and 143.2, 200.5, and 257.7 CNY for flight-intercept traps. Because early warning prioritizes detection, we also calculated cost per detected active site. Under the base scenario, this metric was approximately 932 CNY for lure traps, 1333 CNY for baited logs, and 824 CNY for flight-intercept traps. The lower detection cost estimate for flight-intercept traps reflects their very low assumed unit cost and should not be interpreted alone, because only 63 active flight-intercept sites generated few absolute detections and a narrow operational taxonomic profile. When the numerator was restricted to resolved taxon-level captures, lure traps again had the lowest base-scenario cost per resolved captured individual (50.3 CNY, compared with 1662.5 CNY for baited logs and 2205.0 CNY for flight-intercept traps). Because all values were derived from assumed cost ranges rather than audited accounts, they should be read as supportive sensitivity analyses rather than definitive economic comparisons.

## 4. Discussion

### 4.1. Why Did Lure Traps Dominate Across Nearly All Metrics?

Across the Yunnan monitoring program, lure traps consistently outperformed the other two methods. For routine early warning, the most relevant question is not simply which method catches the largest number of beetles, but which method most reliably detects beetles before severe damage becomes visible. Lure traps were strongest for this primary detection-oriented criterion, and their advantage was also supported by cumulative catch, operational taxonomic output, paired site performance, the more comparable subset, county-clustered models, and the taxonomic resolution sensitivity analysis. For an observational dataset, that degree of consistency matters. It suggests that lure traps were the strongest routine option within this program, even though the exact size of their advantage should still be interpreted as an operational rather than a strictly causal effect.

This result is biologically plausible and consistent with a broad body of literature showing that semiochemical-baited traps actively recruit dispersing adults and therefore extend the effective sampling footprint beyond the immediate intercept surface of the device [1,2,3,12,13,14,15,16,17,18,19,20,21,22,23,24,25]. Recent work has also shown that trap performance depends on approach, contact, retention, and escape processes, not simply on whether a lure is present [12,13,14,19,21]. The strong performance of lure traps in Yunnan should therefore be read as evidence of surveillance effectiveness under a defined monitoring objective, not as a direct estimate of beetle density, emergence, or community composition.

### 4.2. Why Were Baited Logs Weaker, but Still Useful?

Baited logs had much lower detection probability, catch, and operational richness than lure traps, but that does not make them uninformative. Their ecological logic differs from that of semiochemical traps because they provide real or simulated host substrate. As a result, they may still be useful when the monitoring objective includes host use confirmation, immature stage association, or the collection of taxa that respond inconsistently to available synthetic lures [16,27,32]. In practical terms, baited logs are best viewed as a complement to lure traps when evidence from breeding substrate is required, especially because baited-log records can link beetles to local host material in a way that adult trap captures cannot.

At the same time, the province-scale results suggest that baited logs are poorly suited to serve as the first-line routine early warning tool in this program. Their effectiveness likely depends more strongly on the freshness and condition of the host material, their attraction radius is smaller and more variable, and the method is harder to standardize operationally than scheduled lure replacement. Taken together, the results are more consistent with a targeted supplementary role than with use as the backbone of province-wide surveillance [16,32].

### 4.3. Why Did Flight-Intercept Traps Contribute So Little to Routine Early Warning?

Flight-intercept traps produced the lowest abundance and the narrowest operational taxonomic profile in the Yunnan dataset. This result should not be interpreted as evidence that passive interception lacks scientific value. Intercept devices remain useful for biodiversity inventory, faunistic sampling, and exploratory studies of trap behavior [1,2,9,11,18,19]. Their function, however, differs fundamentally from that of active lure systems. Because they depend on insects physically encountering the intercept surface, they are far more sensitive to local flight paths, microhabitat configuration, and device geometry. Under an early warning objective, this passive mechanism is inherently less aligned with the need for high site-level detection probability.

The weak performance observed here suggests that passive interception is not well aligned with the main objective of the Yunnan monitoring program, namely broad-spectrum operational surveillance in healthy standard subcompartments [29]. For routine early warning across a climatically heterogeneous mountain province, a device that samples only the insects whose flight paths happen to cross its intercept surface is at an inherent disadvantage relative to a device that actively attracts dispersers. We therefore treat flight-intercept traps as a specialized complementary method rather than as a direct substitute for lure traps. They may still deserve a place in biodiversity or exploratory surveys, but the present evidence supports only a limited operational role for them in routine early warning deployment.

### 4.4. Landscape Context, Deployment Optimization, and Interpretation Boundaries

A second key result was that monitoring returns peaked within the 1500–2200 m belt and became more variable when devices were concentrated within already well-covered counties. In a mountainous province such as Yunnan, elevation captures several climatic and ecological dimensions at once, including temperature regime, host composition, phenology, and stand condition. Spatial information was used descriptively through the province-scale deployment and active site maps and analytically through county-clustered models. The positive returns were most interpretable in the pine surveillance belt represented by mid-elevation *Pinus yunnanensis* landscapes, although the observational allocation of methods prevents strong taxon-by-county inference. The observed mid-elevation maximum is ecologically plausible and consistent with broader work linking bark- and wood-boring beetle activity to thermal suitability, host stress, and landscape context [4,5,6,7,26,33]. From a management perspective, this pattern offers a practical rule for prioritization when point-level meteorological data are unavailable.

The density result should nevertheless be interpreted cautiously. Our county-level density index was a management proxy rather than a direct measure of local trap interference. The decline or instability in yield under denser deployment is therefore better interpreted as evidence of diminishing returns under clustered allocation than as definitive proof that nearby devices competed mechanically for the same insects. This distinction matters because operational datasets reveal comparative effectiveness under implementation conditions, but they do not always isolate the mechanism behind the pattern [24,26].

### 4.5. Study Strengths, Limitations, and Next Steps

The main strength of this study lies in its province-scale evaluation of surveillance devices under real implementation conditions. Datasets of this kind complement experimental trap trials by showing which methods retain their advantage after variation in revisit frequency, host context, county-level organization, and terrain is allowed to enter the system. The present analysis also moves beyond simple description by combining paired tests, more comparable subset analyses, county-clustered models, and taxonomic resolution sensitivity analyses. A further strength is that the study distinguishes installed sites from active sites, thereby separating nominal deployment coverage from whether deployed devices subsequently generated realized sampling effort.

Several limitations remain. First, taxonomic resolution in the field records was uneven, so richness in the main analysis represents operational taxa rather than a standardized species inventory. Second, lure chemistry was recorded at the active component level, whereas manufacturer-specific release rates, carrier materials, and lure age at every service visit were not consistently available. Third, baited-log bolt length and general pile structure were specified by the technical scheme, but bolt diameter, exact felling-to-deployment interval, and realized exposure duration were not uniformly captured across counties. Fourth, climate was represented by elevation rather than direct weather surfaces. Fifth, revisit timing, lure condition, and local maintenance quality could not be fully standardized after the fact. Sixth, the temporally aggregated dataset did not support a defensible analysis of time to first detection or per-visit detection consistency, even though those metrics would be highly relevant for early warning systems. Seventh, the cost analysis was scenario-based because audited expenditure records were unavailable. Eighth, the transition from installed to active status probably reflected not only ecological conditions but also operational factors such as accessibility, maintenance completion, and local implementation intensity. Finally, because devices were not randomly assigned, even the more comparable subset and county-clustered analyses cannot eliminate residual confounding, and county-level clustering reduced but did not fully remove possible spatial dependence. None of these limitations overturns the main conclusion, but each narrows the range over which the results should be generalized.

Future work should integrate gridded climate covariates, refined host and stand descriptors, audited costs, exact lure release rates, bait log bolt diameter, felling-to-deployment interval and exposure period, per-visit detection histories, and explicit spatial optimization models. It should also evaluate time to first detection, detection consistency across visits, target taxon yield, and taxon-specific geographic distributions across elevation, host group, and county. Trap-level refinements such as color, cup design, anti-escape structures, lure composition, and bycatch reduction should also be tested under Yunnan conditions [12,13,14,15,16,17,18,19,20,21]. The strongest next-generation monitoring architecture will probably combine a provincial lure-trap network for early warning with targeted host-based or destructive follow-up when ecological diagnosis is required.

## 5. Conclusions

The province-wide Yunnan database provides consistent operational evidence that lure traps were the strongest routine early warning tool under the program conditions examined here. This conclusion rests primarily on observed site-level detection rate and is supported by cumulative catch, operational taxonomic coverage, paired site comparisons, common support analysis, adjusted models, taxonomic resolution sensitivity analysis, and secondary cost metrics.

Baited logs should be retained as a targeted complementary method rather than as the primary surveillance device. Their main value lies in host use confirmation, immature-stage acquisition, and diagnostic follow-up when evidence from breeding substrate is needed. Flight-intercept traps may still be useful for faunistic or biodiversity-oriented surveys, but the present dataset supports only a limited role for them in routine early warning.

For Yunnan and comparable mountainous regions, the present data support a practical tiered strategy: prioritize lure traps in the 1500–2200 m pine belt and in county-level coverage gaps with elevated risk, retain baited logs for targeted supplementary deployment where host use confirmation or immature-stage material is required, and reserve flight-intercept traps for specialized exploratory or biodiversity purposes. Because the study was observational, taxonomic resolution was operational, temporal detection histories were not standardized, and the cost assessment was scenario-based, these recommendations should be generalized cautiously. Future monitoring design should integrate gridded climate surfaces, refined stand descriptors, audited costs, exact lure and bait-log specifications, temporal detection metrics, and explicit spatial optimization so that province-scale surveillance can move from extensive deployment toward demonstrably optimized early warning networks.

## Figures and Tables

**Table 1:** Coverage and realized field effort of the three monitoring methods in the Yunnan dataset.

Method	Installed Sites	Active Sites	Installed Units	Collection Events	Mean Elevation (m)
Baited logs	1745	570	4080	3012	1796.0
Flight-intercept traps	361	63	373	164	1850.0
Lure traps	1413	496	4807	6011	1883.0

**Table 2:** Active site performance metrics by monitoring method. Taxon richness and unique taxa refer to operational taxonomic categories rather than standardized species-level richness.

Method	Detection Probability	Total Captured Individuals	Median Catch/Site	Mean Catches Per Collection Event	Mean Operational Taxa Richness/Site	Unique Operational Taxa Recorded
Baited logs	0.21	999	0	0.33	0.26	18
Flight-intercept traps	0.17	44	0	0.27	0.21	6
Lure traps	0.73	8617	3	1.43	1.45	45

## Data Availability

The raw monitoring records used in this study are not publicly available because they were generated through a province-wide operational surveillance program and remain subject to administrative restrictions. Data supporting the findings of this study are available from the corresponding author upon reasonable request and with permission from the data owner. The official technical scheme cited as Ref. [29] is available from the corresponding author upon reasonable request; an English summary of the operational requirements used in this study can be provided with the submission materials if required by the journal.
